# Fate of Bioactive Compounds during Lactic Acid Fermentation of Fruits and Vegetables

**DOI:** 10.3390/foods11050733

**Published:** 2022-03-02

**Authors:** Spiros Paramithiotis, Gitishree Das, Han-Seung Shin, Jayanta Kumar Patra

**Affiliations:** 1 Department of Food Science and Human Nutrition, Agricultural University of Athens, 11855 Athens, Greece; 2 Research Institute of Integrative Life Sciences, Dongguk University, Goyangsi 10326, Korea; gdas@dongguk.edu; 3 Department of Food Science & Biotechnology, Dongguk University, Goyangsi 10326, Korea; spartan@dongguk.edu

**Keywords:** vitamins, GABA, phenolic compounds, organosulfur compounds, bioactive peptides, biogenic amines

## Abstract

Consumption of lactic acid fermented fruits and vegetables has been correlated with a series of health benefits. Some of them have been attributed to the probiotic potential of lactic acid microbiota, while others to its metabolic potential and the production of bioactive compounds. The factors that affect the latter have been in the epicenter of intensive research over the last decade. The production of bioactive peptides, vitamins (especially of the B-complex), gamma-aminobutyric acid, as well as phenolic and organosulfur compounds during lactic acid fermentation of fruits and vegetables has attracted specific attention. On the other hand, the production of biogenic amines has also been intensively studied due to the adverse health effects caused by their consumption. The data that are currently available indicate that the production of these compounds is a strain-dependent characteristic that may also be affected by the raw materials used as well as the fermentation conditions. The aim of the present review paper is to collect all data referring to the production of the aforementioned compounds and to present and discuss them in a concise and comprehensive way.

## 1. Introduction

Lactic acid fermentation has been applied for centuries on substrates of plant and animal origin. The seasonal and geographical diversity of the raw materials results in a great variability of products. The qualitative and quantitative composition of the micro ecosystem that is developed during fermentation; the biotic and abiotic factors that direct it, along with the physicochemical changes of the substrate itself, have been in the epicenter of intensive research for many decades. Nowadays, the interest in lactic acid fermentation has been re-fueled, and its value has again been praised due to the health benefits that their consumption may confer. Indeed, a series of health benefits, including anti-allergic, anti-hypertensive, anti-inflammatory, anti-diarrheal, anti-infection, and anti-aging, as well as prevention and control of chronic diseases such as cardiovascular diseases, type 2 diabetes, obesity, and cancer, has been associated with the consumption of lactic acid fermented commodities. These health benefits have been attributed to the lactic acid bacteria that drive the fermentation as well as to the bioactive compounds that are present in the final product [1,2,3,4,5,6,7,8]. Their presence depends upon the occurrence of the necessary precursor molecules in the raw materials and the capacity of the lactic acid bacteria strains to carry out the required biotransformations.

Lactic acid fermentation of fruits and vegetables is no exception. Indeed, the suitability of fermented fruits and vegetables as probiotic carriers has been adequately exhibited [9,10,11,12,13]. In addition, specific health benefits have been associated with the consumption of specific products, such as the antioxidant, anti-obesity, anti-cancer, anti-hypertensive, and immunomodulatory potential of kimchi [14].

The biotic and abiotic factors that affect the production of vitamins (especially of the B-complex), gamma-aminobutyric acid, bioactive peptides, as well as phenolic and organosulfur compounds during lactic acid fermentation of fruits and vegetables have attracted specific attention over the last decade. In addition, the production of biogenic amines has also been intensively studied due to the adverse health effects that are caused by their consumption. The aim of the present review paper is to collect all relevant information and to present and discuss them in a concise and comprehensive way.

## 2. Vitamins

The role of vitamins in human life and well-being is very important; they facilitate metabolic reactions, including energy-yielding ones, as well as many physiological processes. Depending on their chemical nature, they may be distinguished into water-soluble (B-complex, C) and fat-soluble (A, D, E, K) vitamins. They are considered essential micronutrients since the human body is not able to synthesize the majority of them. Thus, adequate dietary supply is necessary to prevent deficiency. Biofortification, i.e., the utilization of microorganisms capable of producing them, has been proposed as a strategy to improve the vitamin content of certain commodities. This approach is particularly valuable in the case of fermented fruits and vegetables.

The vitamin content of fruits and vegetables has been extensively studied. Fruits are recommended as sources of vitamin C; they also contain vitamin K and carotenoids, and leafy vegetables contain vitamin C, folate, and carotenoids [15]. More specifically, cucumbers and Chinese cabbage contain vitamins C, B1, B2, B11, B3, B6, A, E, and K, with Chinese cabbage appearing to contain more per 100 g. Olives contain vitamins B1, B3, B6, A, E, and K; black olives also contain vitamin C, while green olives also contain vitamin B11. Green olives appear to contain quantitatively more vitamins than black olives, with the exception of vitamin K, where they both contain 1.4 mg/100 g. Vitamins B12 and D seem to be absent from cucumbers, Chinese cabbage, and olives (data from fdc.nal.usda.gov, accessed on 29 July 2021).

Vitamin production by lactic acid bacteria has been in the epicenter of intensive research over the last decade, particularly vitamins of the B-complex and, more specifically, vitamins B2 (riboflavin), B9 (folate), and B12 (cobalamin). Vitamin production seems to be a strain-dependent property. Strains of *Enterococcus faecium*, *Lactococcus lactis* subsp. *lactis*, *Lactobacillus acidophilus*, *Lactiplantibacillus plantarum*, *Limosilactobacillus fermentum*, *Lacticaseibacillus rhamnosus*, *Lm. mucosae*, and *Leuconostoc mesenteroides* have been reported as riboflavin producers [16,17,18,19,20,21,22,23]. Extracellular folate production has been reported for strains of *Streptococcus thermophilus*, *Lb. amylovorus*, *Lp. plantarum*, *Latilactobacillus sakei,* and *Lc. lactis*. [24,25,26,27,28], while cobalamin production has been verified for strains belonging to the lactic acid bacteria species *E. faecium*, *E. faecalis*, *La. casei*, *Furfurilactobacillus rossiae*, *Lm. reuteri*, *Lp. plantarum*, *Loigolactobacillus coryniformis*, *Lm. Fermentum,* and *La. rhamnosus* [29,30,31,32,33,34,35]. The capacity of lactic acid bacteria strains to produce vitamin B1 (thiamine), B3 (niacin), as well as K2 has also been reported [36,37,38].

Although fruits and vegetables and, especially, green vegetables have been recognized as the main sources of folates for humans [39] and certain fruits and vegetables and, especially, dark green vegetables are very good sources of riboflavin [40], the fate of vitamins during lactic acid fermentation has only been marginally studied. Jagerstad et al. [41] reported that folate production takes place during lactic acid fermentation, depending on the starter culture. More accurately, the starter culture, consisting of a mixture of *Lp. plantarum*, *Lc. lactis/cremoris* and *Leuconostoc* sp. strains, was able to produce up to 125 μg/kg 5-CH3-H4 folate during fermentation of a mixture of beetroots, turnips, and onions and 110 μg/kg during fermentation of a mixture of roots consisting of carrots, turnips, parsnips, celeriacs, and onions. Thompson et al. [42] used four *Lp. plantarum* strains to ferment cauliflower, white beans, and their 50:50 mixture and reported a statistically significant increase in riboflavin and folate content. More accurately, after fermentation of the latter at 30 °C for 44 h, riboflavin increased to 75.64–91.60 μg/100 g fresh weight from the 42.83 μg/100 g fresh weight of the unfermented control; folate increased to 48.74–58.82 μg/100 g fresh weight from the 36.84 μg/100 g fresh weight of the unfermented control. In addition, *Lp. plantarum* strain 299 was able to produce vitamin B12, increasing its concentration to 0.048 μg/100 g fresh weight from the 0.029 μg/100 g fresh weight of the unfermented control.

## 3. Gamma-Aminobutyric Acid

**Figure 1:**
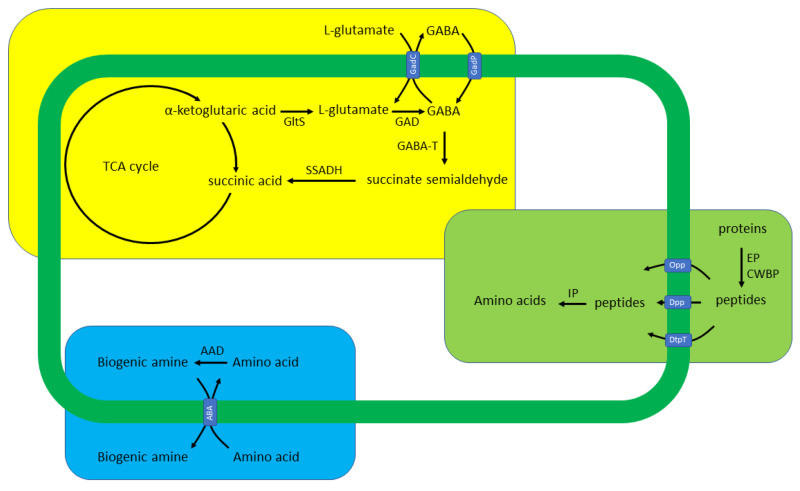
Production of GABA (**yellow box**), bioactive peptides (**green box**), and biogenic amines (**blue box**) by LAB. AAD: amino acid decarboxylase; ABA: amino acid/biogenic amine antiporter; CWBP: cell-wall-bound proteinases; Dpp: peptide (2–9 amino acids) ABC transporter; DtpT: ion linked peptide (2–3 amino acids) transporter; EP: extracellular proteinases; GABA-T: GABA aminotransferase; GAD: glutamate decarboxylase; GltS: glutamate synthase; IP: intracellular peptidases; Opp: oligopeptide (4–18 amino acids) permease; SSADH: succinate semialdehyde dehydrogenase.

The occurrence of gamma-aminobutyric acid (GABA) in plants, microorganisms, and vertebrates has been adequately exhibited. In plants and humans, GABA is mostly associated with signaling functions. Indeed, its role in plant growth and stress response has been established [43,44,45]. In humans, it acts as the major inhibitory neurotransmitter in the central nervous system. The latter has played a decisive role in the ongoing trend of enriching food with this molecule; however, Hepsomali et al. [46] mentioned that although GABA oral intake resulted in various responses [47,48,49], it is still unknown whether brain GABA concentration is increased. On the other hand, it seems to have a different role in microorganisms; it has been associated with resistance to acidic conditions [50] as well as spore germination, at least in *Neurospora crassa* [51] and *Bacillus megaterium* [52]. In lactic acid bacteria, GABA production has been reported as a strain-dependent characteristic. It takes place mostly through L-glutamate decarboxylation since it also contributes to acid resistance through proton consumption [53]. L-glutamate supply may be exogenous through the glutamate/GABA antiporter or endogenous through the activity of glutamate synthase on α-ketoglutaric acid. Then, GABA may be transported extracellularly through the aforementioned antiporter or degraded to succinic acid through GABA aminotransferase and succinate semialdehyde dehydrogenase (Figure 1) Among others, strains belonging to *E. durans*, *La. paracasei*, *La. rhamnosus*, *Lp. plantarum*, *Lb. delbrueckii* subsp. *bulgaricus*, *Lc. lactis* subsp. *lactis*, *Le. buchneri*, *Leu. mesenteroides*, *Leu. pseudomesenteroides*, *Lv. brevis*, *S. salivarius* subsp. *thermophilus,* and *Weissella cibaria* have been reported as GABA-producers [54,55,56].

The amount of GABA synthesized by a plant depends upon several factors, such as variety, type, and severity of biotic and abiotic stresses; however, its occurrence has been characterized as ubiquitous [57]. Indeed, GABA amount may range from the 0.007 mg/g dry weight in an epicarp/mesocarp mixture of apples and the 0.019 mg/g dry weight of chestnuts to the 1.86 mg/g dry weight of mulberries and the 174.30 mg/g fresh weight of *Vitis vinifera* L. cultivar Pinot Noir [58,59,60,61]. Regarding the raw materials mostly used as substrates for lactic acid fermentation, the occurrence of GABA has also been reported. In olives and in extra virgin olive oil, the amount of GABA was cultivar-dependent [62,63]. In the latter case, its amount was less than 0.00014 mg/g. Leaves and roots of Chinese cabbage were reported to contain 4.69 and 7.02 μmol/g dry weight, respectively, accounting for the 8% and 26.86% of total free amino acids, respectively [64]. Finally, fresh cucumbers were reported to contain 105 mg/kg GABA [65].

Current evidence shows that lactic acid fermentation may increase GABA content. Indeed, spontaneously fermented cucumbers were reported to contain 150 mg/kg GABA, with the majority of it being formed during the first day of fermentation [65]. Notably, GABA concentration remained stable throughout the 6-month storage period at 28 °C. In the case of spontaneously fermented olives, GABA was formed only upon monosodium glutamate addition [66]. The amount of GABA formed was proportional to the amount of monosodium glutamate added and irrespective of the osmotic dehydration of olives, which was applied as a pre-fermentation treatment. GABA production was also reported during spontaneous kimchi fermentation [67]. In that study, GABA production took place within the first 25 days of storage at 4 °C, reaching approximately 4 mM; this amount of GABA remained stable until the end of storage (120 days). Analysis of the microecosystem identified strains of *Leuconostoc* spp. and *Lt. sakei* as the GABA producers. Seok et al. [68], Cho et al. [69], and Lee et al. [70] studied GABA production during kimchi fermentation inoculated with GABA producing strains. Seok et al. [68] used *Lactobacillus* sp. strain OPK 2–59 and 5 g monosodium glutamate and managed to produce 18 mg/100 g GABA, a notable increase from the initial amount of 2.84–4.06 mg/100 g. Interestingly, rapid GABA production was observed after the 9th day of storage. In the kimchi produced by the addition of either the GABA-producing strain or monosodium glutamate, the GABA amount at the end of storage (21 d) was less than 6 mg/100 g. Cho et al. [69] analyzed commercially available kimchi and Mukeunjee kimchi products and reported that the GABA content ranged from 1.9 to 12.9 mg/100 g and from 18.2 to 99.0 mg/100 g, respectively. Then, a GABA-producing *Le. buchneri* strain was employed as a starter culture, resulting in kimchi with 61.7 mg/100 g GABA, which was significantly higher than the 8.1 mg/100 g of the spontaneously fermented one. Notably, the sensory scores of the products were comparable. Lee et al. [70] prepared kimchi with the addition of *Lv. zymae* strain GU240 as a starter culture and evaluated the effect of L-glutamic acid, monosodium glutamate, and kelp extract as GABA precursors. Storage took place at −1 °C for 20 weeks. Monosodium glutamate was the most effective GABA precursor. The most rapid increase was observed between weeks 2 and 4, and the maximum GABA concentration reached 120.3 mg/100 g in week 8. Then, it was reduced to the final amount of 95.6 mg/100 g. The GABA content of the kimchi that was prepared without the addition of starter or precursor, as well as the kimchi prepared with only the addition of a starter, was 47 mg/100 g. The addition of kelp extract resulted in the accumulation of 55 mg/100 g GABA, and the addition of L-glutamate resulted in 62.5 mg/100 g. In all cases, maximum GABA concentration was observed in weeks 8 and 10, which was then reduced until the end of storage (week 20).

## 4. Bioactive Peptides

Bioactive peptides are short peptides that, upon release from the parent protein molecule, exert a biological function. Decryption from the parent protein molecule may take place during gastrointestinal digestion or due to the proteolytic activity of microorganisms, such as the LAB that direct a fermentation process. Their occurrence depends on the activity of extracellular and cell envelope proteinases, as well as the peptide transportation capacity into the cell, towards their complete hydrolysis to amino acids [71] (Figure 1). A wide range of biological activities has been described for such peptides, including anti-diabetic, antioxidant, anti-microbial, anti-thrombotic, hypocholesteromic, hypotensive, mineral-binding, opioid, and anti-opioid.

The liberation of bioactive peptides through lactic acid fermentation of protein-rich substrates, such as milk and soy, has been extensively studied. Data on the bioactivity of the peptides released by the lactic acid fermentation of meat, fish, grains, and legumes are also available. Thus, the decryption of such peptides through the application of LAB, such as *E. faecalis*, *Lp. plantarum*, *Lb. helveticus*, *La. casei*, *La. rhamnosus*, *Companilactobacillus farciminis*, *Fructilactobacillus sanfranciscensis*, *Lc. lactis*, *Lb. delbrueckii* subsp. *lactis,* and *Pediococcus acidilactici* single strains [35,72,73,74,75,76,77,78,79,80,81], or microbial consortia [82,83,84,85,86,87,88,89,90], has been reported.

In general, fruits and vegetables are not rich in protein; however, the occurrence of bioactive peptides in some of them has been reported (recently reviewed by Sosalagere et al. [91]). Cucumbers, Chinese cabbage, and green and black olives contain 0.65%, 1.5%, 1.03%, and 0.84% protein, respectively (https://fdc.nal.usda.gov/, accessed on 21 August 2021). In olive seeds, the occurrence of the peptide LLPSY exhibited significant anti-proliferative capacity on prostate cancer cells (PC-3) and breast cancer cells (MDA-MB-468) [92]. Occurrence of bioactive peptides in cucumbers that were raw, acidified, spontaneously fermented, or fermented with the addition of *Lp. pentosus* strain LA0445 was assessed by Fideler et al. [93]. Five peptides with potential anti-hypertensive activity were detected, namely, IPP, LPP, VPP, KP, and RY. KP was present in all cases; the amount in the fermented ones was significantly higher than the rest. Acidified cucumbers also contained KY, a peptide that was not detected in spontaneously fermented ones. The cucumbers that were fermented with the addition of the starter culture contained all five peptides [93].

## 5. Phenolic Compounds

The occurrence of phenolic compounds in plants has been extensively assessed. They are the third-largest group of secondary metabolites, after terpenes and alkaloids; they hold a very important physiological role as they participate in processes such as photosynthesis, respiration, and cell development. Regarding the total phenolic content (TPC) of the fruits and vegetables mostly used as a substrate of lactic acid fermentation, it seems to be rather low; it has been reported to vary between 0.58–1.42 mg GAE/g fresh weight for Chinese cabbage, 0.17 mg GAE/g fresh weight for cucumbers, and 82.29–287.29 mg GAE/100 g for olives [94,95]. Their amount depends upon factors associated with the plant type and variety, cultivation conditions, processing, and storage [96,97]. The interest in phenolic compounds is fueled by the correlation that has been achieved between them and antioxidant capacity as well as the prevention of chronic diseases and inflammation [98].

Based on the fact that lactic-acid-fermented fruits and vegetables consist of two phases, namely, a solid and a liquid one, Ciniviz and Yildiz [99] studied the TPC of both juice and pulp portions of 30 kinds of lactic acid fermented fruits and vegetables. In all cases but two, namely, wild pears pickle and sour grapes pickle, the amount of TPC in juice was higher than the respective in the pulp portion. In the latter, TPC ranged from below detection limit in carrots pickle and white cabbage pickle to 135.39 μg GAE/mg in pinecone pickle, while in the juice portion, it ranged from 16.94 μg GAE/mg in tomatoes pickle to 235.19 μg GAE/mg in pinecone pickle. The most common phenolic acid seemed to be sinapic acid, which was detected in all juice and pulp samples at concentrations ranging from 135.91 mg/L in sour grapes pickle to 236.32 mg/L in sweet long green pepper pickle and from 104.25 mg/kg in white cucumber pickle to 107.43 mg/kg in unripe melon pickle, respectively. Vanillic acid, caffeic acid, and chlorogenic acid were present in all juice samples, ranging from 0.08 mg/L in white cabbage pickle to 31.81 mg/L in carrot pickle, from 30.06 mg/L in unripe melon pickle and chard pickle to 74.61 mg/L in hot pepper pickle, and from 62.21 mg/L in cauliflower pickle to 200.30 mg/L in rock samphire pickle, respectively. 4-hydroxybenzoic acid and p-coumaric acid were not detected in any sample.

The fate of phenolic compounds during lactic acid fermentation has only been marginally studied. The mode by which lactic acid fermentation may increase the TPC of the raw materials is either through the lysis of the cell wall of the plant cells with concomitant facilitation of their release from the vacuole, in which they are mainly localized, or by enzymatic conversion of their glycosides into their aglycone form [100]. The latter may take place through β-glycosidase activity, which several lactic acid bacteria strains have exhibited [101,102]. Indeed, several *Lp. plantarum* strains have been reported to hydrolyze oleuropein, which is the main phenolic glucoside of olives [103]. More accurately, an initial action of β-glycosidase, followed by an esterolytic activity on the aglycone moiety, has been reported to produce olenoic acid and hydroxytyrosol [100]. Moreover, through the production of phenolic acid decarboxylases, some *Lp. plantarum* strains may decarboxylate phenolic acids [104,105].

A wide range of phenolic compounds have been reported to occur in the brine or the flesh of fermented olives, including apigenin, apigenin-7-O-glucoside, caffeic acid, p-coumaric acid, cyanidin-3-O-glucoside, cyanidin-3-O-rutinoside, ferulic acid, p-hydroxybenzoic acid, hydroxytyrosol, luteolin, luteolin-4-O-glucoside, luteolin-7-O-glucoside, protocatechuic acid, pyrocathecol, rutin, tyrosol, vanillic acid, vanillin, and verbascoside [106,107,108,109,110]. Their fate during fermentation depends upon the cultivar, the addition and type of starter culture, brine composition, as well as fermentation temperature and time [107,108,109]. Indeed, proper arrangement of the aforementioned conditions may result in the complete decomposition of oleuropein and an increase in hydroxytyrosol, tyrosol, p-coumaric acid, vanillic acid, caffeic acid, verbascoside, and ferulic acid [107,109,110]. Initial concentration increase has also been reported for apigenin-7-O-glucoside, luteolin-4-O-glucoside, and luteolin-7-O-glucoside, which was followed by a decrease until the end of fermentation [110].

In the case of kimchi, Park et al. [111] reported that over-ripened kimchi contained more TPC than short-term fermented ones. Park et al. [112] reported that the TPC of mustard kimchi increased during the first two months to 482.4 mg GAE/g extract powder but then decreased during the third month to 475.3 mg GAE/g extract powder, which had no statistically significant difference from the control. Regarding the specific phenolic compounds assessed, the amount of caffeic acid increased throughout the three months of fermentation; the amount of naringin, catechin gallate, and epigallocatechin gallate initially increased, but after three months of fermentation, their amount was less than that of the control. The amount of chlorogenic acid and epicatechin gallate decreased throughout the three-month fermentation compared to the control; p-coumaric acid and gallocatechin gallate were only detected after one month of fermentation, and catechin was only detected after one and two months of fermentation. Epicatechin and rutin were present in the control, and their amount increased after two months of fermentation. However, they were not detected after three months of fermentation. Finally, gallic acid and epigallocatechin were not detected to the control and throughout fermentation. Oh et al. [113] studied the TPC of Dolsan leaf mustard kimchi and reported that the TPC of leaves decreased during the first 21 days of fermentation but then increased to the initial amount of ca. 100 mg GAE/100 g. On the contrary, the TPC of stems gradually increased from the initial ca. 40 mg GAE/100 g to a final of ca. 110 mg GAE/100 g. A novel insight was provided by Jung et al. [114]. In that study, the increase of TPC over the 24-day fermentation of kimchi made of young Chinese cabbage was reported. However, the initial TPC and the TPC at the end of fermentation were determined at 83.2 and 102.5 mg GAE/100 g, respectively, when the young Chinese cabbage was cultivated using nature-friendly composts. These amounts of TPC were higher than 63.2 and 98.2 mg GAE/100 g, respectively, when the young Chinese cabbage was cultivated using general composts and higher than 57.9 and 81.0 mg GAE/100 g, respectively, when the young Chinese cabbage was cultivated using chemical fertilizers.

Ciska et al. [115] and Kapusta-Duch et al. [116] studied the TPC of sauerkraut. The first study reported that sauerkraut extract contained 8.25 mg/g TPC while white cabbage contained 5.72 mg/g. In white cabbage, only esterified phenolic acids were detected, with sinapic acid being the most prevalent (278 μg/g). On the contrary, apart from esterified phenolic acids, their glycosides were also detected in the sauerkraut extract. As in the previous case, sinapic acid was the prevalent one, with 20 μg/g being quantified as esterified acid and 84 μg/g as its glycoside. Kapusta-Duch et al. [116] assessed the effect of package type, namely, low-density polyethylene and metalized polyethylene terephthalate with polyethylene bags, on the TPC content during four months of chilled storage of white sauerkraut. It was revealed that package type had no effect on the TPC levels as in both cases, the reduction was at ca. 12% and 20% after three and four months of storage, respectively.

The fate of phenolic compounds has also been assessed in less known regional lactic acid fermented fruit and vegetable products, such as African nightshade leaves and kiwi fruit. In the first case, the effect of fermentation that was carried out at 37 °C for 3 days on the phenolic profile of the product was strain-dependent [117]. For example, fermentation with *Lp. plantarum* strain 75 resulted in an increase of the amount of gallic acid, vanillic acid, 2,5 dihydroxybenzoic acid, p-coumaric acid, and ellagic acid, as well as the flavonoids assessed, namely, catechin, quercetin, and luteolin. On the contrary, fermentation with *Leu. pseudomesenteroides* strain 56 resulted in an increase of the amount of ellagic acid and quercetin and a decrease in gallic acid, caffeic acid, vanillic acid, 2,5 dihydroxybenzoic acid, p-coumaric acid, ferulic acid, and catechin. The effect of fermentation at 37 °C for 28 h by *Lp. plantarum* on the phenolic profile of kiwifruit pulp was studied by Zhou et al. [118]. The TPC increased after the 21st hour of fermentation. The amount of protocatechuic acid, esculetin, and p-coumaric acid was increased due to the fermentation, while the amount of gallic acid, chlorogenic acid, catechin, and epicatechin was decreased.

## 6. Organosulfur Compounds

Vegetables of the family Brassicaceae are very rich in organosulfur compounds in general and glucosinolates in particular. Among others, this family includes all types of cabbage and mustard greens, which are very important raw materials for lactic acid fermentation.

Glucosinolates are secondary metabolites, the stability of which depend upon their contact with myrosinase, a β-thioglucosidase that catalyzes its decomposition. In intact plant cells, they are spatially separated; however, upon conditions that compromise plant tissue integrity, such as infection by herbivores and phytopathogenic microorganisms, the substrate and the enzyme are mixed, leading initially to the formation of the unstable thiohydroximate-O-sulfonate and β-D-glucose. The fate of the former depends on the nature of the side chain present in the glucosinolates molecule as well as the environmental conditions. Especially regarding the latter, neutral pH favors the formation of isothiocyanates while acidic pH in the presence of ferrous ions and epithiospecifier protein favors the formation of nitriles [119,120,121]. The physiological role of glucosinolates and their breakdown products, especially isothiocyanates, against biotic stresses has been verified [122]. Their concentration depends upon plant species, variety, and tissue, as well as environmental conditions and agricultural practices [123]. Although this response may indicate a possible role in abiotic stresses as well, this has not been yet clarified [122].

The interest on glucosinolates and their breakdown products results from their biological activity; many of them have exhibited anti-bacterial, anti-fungal, and anti-proliferative activity against human cancer cells [124]. The biotransformations of glucosinolates during lactic acid fermentation of *Brassica* vegetables have been studied to some extent. In general, fermentation seems to facilitate glucosinolates decomposition and an increase of the concentration of the breakdown products, the type and concentration of which are related to the glucosinolate type and concentration in the raw material as well as the capacity of the microbial strains that drive the fermentation.

Glucosinolate decomposition during fermentation has been exhibited in the case of sauerkraut [125,126,127,128] and has been primarily attributed to the shredding of the cabbage that precedes fermentation and secondarily to hydrolysis by lactic acid bacteria [129,130]. Interestingly, the capacity of LAB to produce nitriles instead of reduced glucosinolates, which were produced by Enterobacteriaceae, was highlighted by Mullaney et al. [129].

Ciska and Pathak [131] reported that glucobrassicin and sinigrin were the most abundant glucosinolates in the shredded cabbage used for fermentation. Ascorbigen, indole-3-carbinol, and indole-3-acetonitrile were identified as degradation products of the former, while allyl isothiocyanate, allyl cyanide, and 1-cyano-2,3-epithiopropane were identified as degradation products of the latter.

Penas et al. [132] highlighted the importance of the starter culture, cabbage cultivar, and fermentation conditions on the volatile glucosinolate breakdown products. Iberin, iberin nitrile, allyl isothiocyanate, sulforaphane, and allyl cyanide were detected, with the latter being the most abundant, ranging from 65 to 75 μmol/100 g DM. Ascorbigen has been reported as the most abundant glucosinolate degradation product in sauerkraut [126,128,131,133]. Ciska and Pathak [131] reported that ascorbigen concentration could be as high as 14 μmol/100 g. Palani et al. [126] quantified ascorbigen at the end of fermentation at 13 μmol/100 g FW. Indole-3-acetonitrile was also present at the end of fermentation at 4.52 μmol/100 g FW. The concentration of both compounds decreased during storage at 4 °C. These results concur with the ones presented by Penas et al. [133] but only as far as the decrease of ascorbigen concentration is concerned; the concentration of indole-3-carbinol and indole-3-acetonitrile was stable throughout three-month storage at 4 °C. Ascorbigen was also reported by Ciska et al. [128] as the main glucobrassicin breakdown product in sauerkraut, which, at the end of fermentation, reached 9.59 μmol/100 g. The concentration of indole-3-acetonitrile and 3,3′-diindolylmethane also increased during fermentation to 0.036 and 0.0099 μmol/100 g, respectively. After 17 weeks of storage at 5 °C, the concentration of ascorbigen decreased to 8.59 μmol/100 g, but the respective of indole-3-acetonitrile and 3,3′-diindolylmethane increased to 0.057 and 0.0187 μmol/100 g, respectively. Regarding the decomposition products of aliphatic and aryl glucosinolates, an increase in the concentrations of allyl isothiocyanate, but-3-enyl isothiocyanate, 3-(methylthio) propyl isothiocyanate, 1-cyano-3-(methylthio) propane, 4-(methylthio) butyl isothiocyanate, 3-(methylsulfinyl) propyl isothiocyanate, 1-cyano-3-(methylsulfinyl) propane, 4-(methylsulfinyl) butyl isothiocyanate, and 2-phenethyl isothiocyanate was reported at the end of fermentation. Isothiocyanates increased during the first days of fermentation, reaching their peak on the 4th day and then decreasing. Allyl isothiocyanate was the most abundant breakdown product, with 2.848 μmol/100 g, followed by 3-(methylsulfinyl) propyl isothiocyanate, with 2.453 μmol/100 g. The concentration of all compounds decreased after 17 weeks of storage at 5 °C, with the exception of 1-cyano-3-(methylthio) propane, which increased [128].

The significance of starter cultures in the fate of glucosinolates during the fermentation of broccoli puree and juice was highlighted by Cai et al. [134], Ye et al. [135], and Xu et al. [136]. Ye et al. [135] studied the effect of lactic acid fermentation of autoclaved broccoli puree using five *Lp. plantarum* and 2 *Leu. mesenteroides* strains on the glucosinolate content. In general, a total of 10 glucosinolates have been detected in broccoli florets, namely, glucoalyssin, glucobrassicanapin, glucobrassicin, 4-hydroxy glucobrassicin, 4-methoxy glucobrassicin, glucoerucin, glucoiberin, glucoraphanin, neoglucobrassicin, and progoitrin [137,138,139]. A strain-dependent increase in the concentration of glucoraphanin, glucoiberin, and progoitrin to 29.0–236.5, 16.1–56.2, and 24.5–65.9 μg/g, respectively, from the initial trace levels, was reported. Notably, the maximum amounts were achieved by *Lp. plantarum* strain F1. Xu et al. [136] reported an increase of glucoraphanin, a decrease of gluconapin, glucoerucin, 4-hydroxy-glucobrassicin, and neoglucobrassicin, and no statistically significant change in glucobrassicin and 4-methoxy-glucobrassicin when juice made of broccoli florets was fermented with two *P. pentosaceus* strains at 37 °C for 36 h, in a strain-dependent manner. Finally, Cai et al. [134] reported that the preheating of broccoli florets at 65 °C for 3 min increased the concentration of sulforaphane, a glucoraphanin decomposition product, from the initial 806 to 3536 μmol/kg DW. Fermentation by a mixture of *Lp. plantarum* and *Leu. mesenteroides* strains at 30 °C for 15 h further enhanced sulforaphane concentration to 13,121.3 μmol/kg DW, most likely by facilitating the release and accessibility of glucoraphanin for decomposition.

Endogenous myrosinase inactivation and concomitant sinigrin retention after lactic acid fermentation of Indian mustard leaves at 22 °C for 7 d were assessed by Nugrahedi et al. [140]. Although oven heat treatment at 35 °C for 2.5 h and microwave treatment at 180 W for 4.5 min effectively reduced myrosinase activity, complete inactivation was achieved by microwave treatment at 900 W for 2 min, leading to the production of sayur asin with the sinigrin concentration of 11.4 μmol/10 g d.m. Mustard leaves are also the basic ingredient for the production of mustard leaf kimchi. Oh et al. [113] studied the fate of glucosinolates during the fermentation of mustard leaf kimchi at 0 °C for 35 d. Sinigrin, gluconapin, glucobrassicin, and glucoraphanin were detected at day 0 in both mustard leaves and stems; gluconasturtiin was only detected in leaves, while glucoiberin was only detected in stems. Reduction of the total amount of glucosinolates was evident throughout fermentation in both leaves and stems, which is mainly assigned to the reduction of sinigrin concentration, which was the most abundant glucosinolate; it was quantified at 21.43 and 22.47 mg/100 g at day 0 and 12.5 and 10.4 mg/100 g at day 35 in leaves and stems, respectively.

## 7. Biogenic Amines

Biogenic amines are compounds formed through the amination and transamination of aldehydes and ketones or the decarboxylation of amino acids. Their physiological role is very important. In plants, the role of polyamines in cell division [141], root growth [142,143,144,145], and vegetative propagation [146,147], as well as flower and fruit development [148,149,150,151,152,153,154], has been exhibited. Moreover, their role in abiotic and biotic stress responses has also been claimed [155,156,157,158,159,160,161,162,163,164,165,166,167,168]. Similarly, the contribution of cadaverine and dopamine to signaling stress response as well as plant growth and development has been reported [169,170]. In addition, tyramine and tryptamine are produced as defensive substances against aggressors [171] and serve as precursors for the production of alkaloids [172] and melatonin [173], respectively.

Regarding microbial physiology, the role of biogenic amines in gene expression [174,175], protection against oxidative stress [176,177,178,179], biofilm formation [180,181], signaling [182,183], and virulence [184,185] has been indicated. From a fermentation perspective, the most important role seems to be the response mechanism against acid stress. This mechanism involves a membrane antiport, which couples amino acid uptake with biogenic amine excretion, and intracellular amino acid decarboxylases, which decarboxylate the inserted amino acid with simultaneous proton consumption (Figure 1). Then, the amine is excreted, and ATP synthesis through proton motive force is directed [186,187]. Such mechanisms have been reported for histidine/histamine, lysine/cadaverine, ornithine/putrescine, and tyrosine/tyramine [186,188,189].

Based on the above, the occurrence of biogenic amines in plant tissues seems justified even without microbial infection and proliferation. Indeed, several studies have reported their presence in nonfermented fruits, vegetables, nuts, legumes, and cereals (reviewed by Sanchez-Perez et al. [190]). Putrescine seems to be commonly occurring and may be accompanied by tyramine, cadaverine, spermine, spermidine, and even histamine [190,191]. This is also the case for white cabbage, Chinese cabbage, and cucumbers, which are commonly used as raw materials for lactic acid fermentation [192,193,194,195,196,197,198]. On the other hand, the occurrence of biogenic amines has not been reported in the flesh of fresh olives at any ripeness stage [199].

In Table 1, the outcome of studies on the quantitative determination of biogenic amines in fermented fruits and vegetables is summarized. Kimchi seems to be the most studied product, most likely due to the variety of raw materials employed, which results in a large diversity of products [14]. Regarding the mean values that have been reported, the highest were 16.81 mg/kg for agmatine [200], 14.3 and 49.8 mg/100 g for cadaverine and histamine, respectively [201], 4.4 mg/kg for phenylethylamine [202], 334.64 mg/kg for putrescine [203], 31.30 mg/kg for spermine [204], and 24.6, 32.3 and 78.0 mg/kg for spermidine, tryptamine and tyramine, respectively [205]. Regarding the highest amounts, agmatine was reported at 86.0 mg/kg [200], phenylethylamine and tyramine at 15.75 and 181 mg/kg, respectively [204], putrescine at 982.32 mg/kg [203], while for cadaverine, histamine, spermidine, spermine, and tryptamine were reported at 155, 535, 8.8, 12.1, and 11.4 mg/100 g, respectively [201].

The level of biogenic amines in sauerkraut was lower than that of kimchi, with the exception of tyramine (Table 1). In the latter case, Kalac et al. [209] analyzed 53 samples of Czech sauerkraut and reported the mean amount at 235 mg/kg and the highest amount at 951 mg/kg. Reports on the biogenic amine content of fermented cucumbers and olives are generally lacking in the literature. Based on the available data (Table 1), fermented cucumbers seem to contain more biogenic amines than fermented olives but less than kimchi and sauerkraut. Regarding fermented olives, they seem to contain less biogenic amines than kimchi, sauerkraut, and fermented cucumbers. Regarding the rest of the fermented fruits and vegetables, the high amounts of agmatine (6.73 mg/kg) and spermidine (74.58 mg/kg) detected in champignon, of histamine (55.60 mg/kg) in white cabbage, of phenylethylamine (9.41 mg/kg), putrescine (252.58 mg/kg), and tyramine (166.58 mg/kg) in Brussels sprouts, of cadaverine (119.42 mg/kg) in broccoli, of spermine (49.63 mg/kg) in pumpkin, and of tryptamine (21.58 mg/kg) in cauliflower should be noticed (Table 1).

The increase in the amount of biogenic amines in fermented foods compared to that of raw materials has been correlated with the microbiota that drive the fermentation. Indeed, the capacity of lactic acid bacteria to decarboxylate amino acids has been adequately exhibited [211]. However, it should be noted that this is a strain-dependent property. Thus, the haphazard nature of the biogenic amine content of spontaneously fermented fruits and vegetables is indicated. On the other hand, qualitative and quantitative control of biogenic amine production is an option that is offered when lactic acid fermentation is performed with the addition of starter cultures.

In the case of sauerkraut, the effect of raw materials on the production of biogenic amines has been highlighted by Majcherczyk et al. [212] and by Satora et al. [213]. In the latter study, eight cabbage varieties were employed to make sauerkraut through spontaneous fermentation; statistically significant differences in tyramine, histamine, cadaverine, putrescine, and tryptamine content were reported. Interestingly, a positive correlation between biogenic amine production and yeast presence was reported. The contribution of yeasts in the accumulation of biogenic amines is already known in products of alcoholic fermentation, such as wine [214]. The addition of ingredients that have an organoleptic impact, such as onion and caraway, affected the accumulation of some biogenic amines; the most pronounced effect was the reduced amounts of cadaverine and tyramine at the end of the 14-day fermentation period [212]. On the other hand, at the end of the 12-month storage at 4 °C, the sauerkraut made at 18 °C, with the addition of onion, accumulated significantly less cadaverine and phenethylamine compared to the control [212]. During spontaneous sauerkraut fermentation, the accumulation of biogenic amines seems to be affected by the aforementioned parameters, along with fermentation temperature and time. Indeed, Rabie et al. [215] reported an accumulation of histamine, tyramine, putrescine, and cadaverine after 10 days of fermentation at 15 °C, while Majcherczyk and Surowka [212] reported accumulation of cadaverine, tryptamine, and tyramine during fermentation at 18 °C for 14 d and of putrescine and tryptamine during fermentation at 31 °C for 14 d. Similarly, accumulation during sauerkraut storage seems to be affected by the same parameters as above. Regarding the effect of cultivar, the accumulation pattern seems to be affected by the cabbage cultivar [216,217,218]; however, no details were provided on the capacity of the members of the microecosystem to perform amino acid decarboxylation. The addition of onion seemed to prohibit the accumulation of cadaverine and phenethylamine but only in sauerkraut fermented at 18 °C and not 31 °C [212]. The paramount effect of a lactic acid bacteria strain’s decarboxylating capacity in the accumulation of biogenic amines has also been adequately exhibited. Indeed, statistically significant differences in the accumulation during storage were observed and assigned to the *Lp. plantarum* and *Leu. mesenteroides* strains that were used as inocula [219]. In addition, suppression of biogenic amine accumulation during fermentation and storage through inoculation with *Lp. plantarum*, *Lt. curvatus,* and *La. casei* was reported by Rabie et al. [215]. Interestingly, the importance of the interaction between the selected starter culture and the cabbage cultivar was highlighted by Kalac et al. [216] and Spicka et al. [220].

In the case of kimchi, the accumulation of biogenic amines during fermentation of four kimchi types, namely, Pa, Gat, Kkakdugi, and Chonggak, has been assessed. Lee et al. [204] prepared Pa and Gat kimchi and studied the effect of myeolchi-aekjeot, a fermented anchovy sauce, the addition of which has been correlated with increased biogenic amine content [202,221,222]; tyramine-producing *Lv. brevis* strains and *Lp. plantarum* strains were unable to produce biogenic amines. During fermentation of Pa and Gat kimchi, the amount of tryptamine and histamine was reduced. During Pa fermentation, accumulation of tyramine, putrescine, and cadaverine in all experimental cases was noted. Spermine was accumulated only in some cases, while β-phenylethylamine and spermidine amounts were stable throughout fermentation. During Gat fermentation, only the cadaverine amount remained unchanged throughout the fermentation, and the accumulation of tyramine, β-phenylethylamine, putrescine, and spermidine was recorded. Spermine was also accumulated but only in some cases. In general, the addition of myeolchi-aekjeot and the tyramine-producing *Lv. brevis* strain enhanced biogenic amine accumulation, with the exception of spermine. Jin et al. [203] prepared Kkakdugi and Chonggak kimchi and studied the effect of myeolchi-aekjeot and saeu-jeotgal, a fermented shrimp product, the utilization of which has also been correlated with increased biogenic amine levels [221] in tyramine-producing *Lv. brevis* strains and *Lp. plantarum* strains. During fermentation of both products, the histamine amount decreased and the spermidine amount increased. In the case of Kkakdugi, tyramine was accumulated only in the samples inoculated with *Lv. brevis*, the putrescine amount slightly increased only in the uninoculated sample and the one inoculated with *Lv. brevis* JCM 1170, and cadaverine accumulated in all samples with the exception of the one that did not contain myeolchi-aekjeot, saeu-jeotgal, and inoculum. The latter sample was the only one in which spermine content increased, while in the rest, it was decreased. In the case of Chonggak, cadaverine remained stable in all samples but seemed to increase in the sample that did not contain the fermented fish condiments and inoculum; the tyramine amount increased in all samples with the exception of the one inoculated with *Lp. plantarum*, and cadaverine was accumulated only in the sample prepared with the addition of the fish condiments but without inoculum. In the same sample, along with the samples inoculated with *Lv. brevis*, the amount of spermine decreased. Combining the studies of Lee et al. [204] and Jin et al. [203], it can be concluded that the accumulation of biogenic amines could not always be predicted through the addition of ingredients that have been correlated with increased biogenic amine content (fish condiments) of starter cultures with known capacities. This indicates the existence of additional parameters that, at least in some cases, may affect biogenic amine accumulation. Since biogenic amine accumulation and decomposition are strain-dependent properties, it can be hypothesized that the native microbiota, and especially the proportion of which that manages to participate in the developing microecosystem, may be this additional parameter.

In the case of fermented cucumbers, biogenic amine accumulation during fermentation was assessed by Alan [223]. In the latter study, gherkin fermentation was performed spontaneously or with the addition of *Lp. plantarum*, *Lp. pentosus,* or *Lp. paraplantarum* strains as starter cultures. Spermidine was not detected in any experimental case. On the contrary, putrescine, cadaverine, histamine, and tyramine were detected, and their amount was strain-dependent. More accurately, spontaneously fermented gherkins contained an equal amount of putrescine with the one started with *Lp. plantarum* strain 49, less than the one started with *Lp. plantarum* strain 51, and more than the ones started with *Lp. plantarum* strain 13, *Lp. pentosus* strain 2, and *Lp. paraplantarum* strain 16. Cadaverine and histamine were not accumulated in the gherkins started with all three *Lp. plantarum* strains, but larger amounts were detected in the gherkins started with *Lp. pentosus* strain 2 and *Lp. paraplantarum* strain 16 compared to the spontaneously fermented ones. Finally, an equal amount of tyramine was accumulated in the gherkins started with *Lp. plantarum* strain 13 comapred with spontaneously fermented ones and larger amounts in the ones started with *Lp. plantarum* strains 49 and 51, *Lp. pentosus* strain 2, and *Lp. paraplantarum* strain 16.

The fate of biogenic amines during the fermentation of olives of the Manzanilla cultivar was assessed by Garcia-Garcia et al. [224]. Putrescine, tryptamine, β-phenylethylamine, spermidine, spermine, histamine, and agmatine were not detected during storage at 15, 20, and 28 °C for 12 months. Only cadaverine and tyramine were accumulated. The former was produced only at 20 and 28 °C, and the production rate increased after 7 and 5 months, respectively. Washing was correlated with increased production. Tyramine production followed a similar trend, with the exception that accumulation also occurred during storage at 15 °C.

## 8. Conclusions

The functional potential of lactic acid fermented fruits and vegetables relies on the interplay between the quality of the raw materials and the capacity of the microbial consortium to carry out certain biotransformations. The former depends on the type and variety of the raw materials, climatic conditions, and agricultural practices, as well as the occurrence and conditions of processing and storage. On the other hand, the production of bioactive compounds by microorganisms is a strain-dependent characteristic that also depends on the fermentation temperature and time. Thus, optimization of the functional potential requires a thorough study of all the aforementioned parameters. Although a lot of information is available in some cases, e.g., the production of biogenic amines, the trophic relationships within the microecosystem are very complex, and, thus, further study is still necessary to enable practical recommendations.

## Figures and Tables

**Table 1:** Occurrence of biogenic amines in fermented fruits and vegetables.

Product.	N	AGM	CAD	HIS	PHE	PUT	SPD	SPM	TRP	TYR
Kimchi types										
Kkakdugi kimchi ^1^	5		27.28 (54.44) [<0.1–124.60]	55.94 (44.45) [18.75–127.78]	3.61 (6.55) [<0.1–15.24]	334.64 (427.97) [10.85–982.32]	9.40 (6.68) [<0.1–16.76]	1.03 (1.31) [<0.1–3.10]	<0.1	25.42 (29.59) [2.97–76.95]
Chonggak kimchi ^1^	5		64.08 (65.51) [2.00–148.50]	58.73 (46.02) [8.24–131.20]	0.78 (1.23) [<0.1–2.80]	269.07 (349.93) [3.89–853.70]	9.06 (2.99) [6.10–14.00]	6.23 (8.79) [<0.1–20.74]	9.02 (9.86) [<0.1–23.70]	8.49 (6.80) [0.79–18.70]
Pa kimchi ^2^	13		44.07 (42.85) [<0.1–123.29]	155.85 (139.26) [8.67–386.03]	1.77 (2.04) [<0.1–5.97]	78.79 (79.00) [<0.1–158.33]	9.91 (4.89) [2.32–18.74]	21.75 (8.94) [<0.1–33.84]	6.99 (5.74) [<0.1–14.92]	66.88 (74.91) [<0.1–181.10]
Gat kimchi ^2^	13		20.5 (18.52) [2.12–48.60]	58.44 (75.77) [3.30–232.10]	3.44 (4.30) [<0.1–15.75]	134.96 (220.53) [1.89–720.82]	20.31 (6.35) [12.26–28.49]	31.30 (22.35) [<0.1–58.57]	11.22 (8.23) [<0.1–26.74]	76.15 (65.91) [1.28–149.77]
Cabbage kimchi (Korean) ^3^	10		15.2 [3.6–44.9]	50.0 [3.4–142.3]	3.0 [nd–6.8]	69.7 [15.1–44.9]	12.0 [7.8–16.5]	2.4 [1.2–3.7]	12.3 [2.3–22.6]	49.4 [9.7–118.2]
Cabbage kimchi (Chinese) ^3^	10		12.5 [3.7–31.0]	2.7 [0.6–8.5]	4.4 [2.1–6.7]	70.6 [16.0–240.4]	11.9 [7.7–15.2]	2.1 [nd–3.7]	12.1 [2.4–20.0]	35.1 [10.7–76.0]
Baechu kimchi ^4^	14		18.0 (18.6) [nd–45.0]			64.6 (73.1) [nd–245.9]	7.8 (5.8) [nd–14.9]		15.0 (16.1) [tr–43.9]	44.0 (35.8) [tr–103.6]
Kkakduki ^4^	5		31.0 (26.8) [nd–56.2]			30.3 (21.7) [nd–51.6]	6.7 (10.3) [nd–21.8]		14.2 (6.1) [5.5–18.6]	4.8 (5.6) [nd–10.8]
Chonggak kimchi ^4^	3		28.6 (49.5) [nd–85.7]			10.7 (10.2) [nd–20.3]	nd		7.3 (6.9) [2.3–15.2]	45.4 (21.9) [20.2–58.1]
Matkimchi ^4^	4		30.5 (35.3) [nd–64.2]			72.1 (27.7) [40.2–104.6]	5.2 (4.5) [nd–10.8]		32.3 (26.9) [nd–60.5]	78.0 (22.0) [54.3–105.1]
Ripened Baechu kimchi ^4^	4		55.7 (18.9) [28.0–63.3]			110.3 (44.4) [57.2–154.6]	24.6 (34.0) [tr–74.8]		5.5 (6.7) [nd–13.6]	46.6 (39.6) [nd–95.6]
Baek kimchi ^4^	3		18.5 (7.1) [11.5–25.6]			20.7 (18.9) [1.9–39.6]	0.8 (0.9) [nd–1.7]		tr	36.4 (28.6) [7.8–64.9]
Super market kimchi ^5^	20	<0.1	14.3 (9.2) [<0.1–155]	49.8 (32.5) [<0.1–535]	<0.1	2.06 (1.33) [<0.1–7.3]	0.65 (0.51) [<0.1–8.8]	1.96 (1.31) [<0.1–12.1]	1.0 (1.05) [<0.1–11.4]	0.46 (0.48) [<0.1–4.2]
Retail market kimchi ^5^	17	<0.1	1.59 (1.44) [<0.1–4.8]	5.59 (4.57) [<0.1–18.6]	<0.1	0.67 (0.79) [<0.1–5.1]	0.48 (0.52) [<0.1–8.2]	<0.1	<0.1	0.40 (0.65) [<0.1–3.5]
Cabbage kimchi ^6^	20		8.3 [0.9–39.8]	6.3 [nd–21.8]	0.5 [nd–2.0]	47.6 [2.3–148.6]	2.9 [nd–6.7]	1.1 [nd–5.1]	11.6 [nd–74.8]	8.3 [1.1–27.9]
Kimchi ^7^	ud	16.81 (30.81) [<0.14–86.00]	63.51 (69.81) [1.13–193.00]	18.53 (28.07) [<0.09–74.94]	2.59 (1.27) [0.94–4.50]	208.70 (186.90) [2.25–475.06]	10.35 (4.49) [5.55–18.25]	1.38 (0.68) [0.56–2.38]	4.75 (9.41) [<0.29–24.88]	59.11 (44.70) [1.25–98.31]
Sauerkaut										
Czech ^8^	53		64.8 (56.8) [1.9–293]	12.1 (31.6) [nd–229]		181 (108) [2.8–529]	8.2 (7.3) [nd–47.0]		4.6 (9.0) [nd–36.5]	235 (213) [nd–951]
Austrian ^8^	10		43.4 (21.0) [19.3–77.4]	2.1 (2.4) [nd–8.0]		179 (80.2) [51.0–295]	6.5 (5.5) [nd–16.9]		2.4 (3.2) [nd–7.7]	130 (71.3) [14.0–214]
Household ^8^	29		29.8 (23.0) [nd–82.7]	4.6 (6.8) [nd–32.4]		87.3 (72.2) [4.3–260]	10.2 (7.5) [nd–28.3]		4.7 (7.9) [nd–28.1]	117 (113) [nd–384]
Sterilized ^8^	29		45.5 (40.1) [6.9–167]	4.9 (6.4) [nd–26.4]		132 (81.5) [18.4–359]	6.8 (4.0) [nd–15.2]		7.2 (10.2) [nd–37.5]	134 (90.4) [26.3–345]
Sauerkraut ^9^			3.9	1.5	tr	9.2	0.5	0.2	nd	4.8
Cucumbers										
Fermented cucumber brine ^10^	1	3.19	45.11	3.07	1.83	61.70	21.16	9.77	7.37	5.24
Pickled cucumbers ^11^	11		nd	nd		4.5 (5.0)		2.9 (4.2)		0.7 (0.8)
Cucumber ^7^	ud	0.65 (1.05) [<0.14–2.88]	82.14 (44.03) [39.60–179.19]	31.54 (7.43) [18.50–40.85]	3.10 (1.64) [1.15–6.31]	171.30 (55.46) [103.13–286.88]	8.08 (3.77) [2.25–14.05]	1.28 (0.71) [0.56–2.65]	14.49 (5.82) [6.00–22.88]	62.24 (20.12) [28.38–86.75]
Olives										
Fermented olive brine ^10^	1	0.81	15.62	1.14	1.78	42.94			0.51	6.93
Olives ^7^	ud	<0.14	2.54 (3.28) [<0.06–6.25]	1.71 (1.58) [<0.09–3.13]	0.23 (0.40) [<0.35–0.70]	17.13 (15.00) [5.75–34.13]	1.21 (0.85) [0.25–1.88]	1.58 (0.85) [0.60–2.13]	3.33 (5.77) [<0.29–10.00]	2.56 (1.29) [1.75–4.05]
Olives ^12^	7		0.80 (0.00) [<0.4–0.8]	nd		5.00 (2.96) [<0.5–7.8]				nd
Various products										
Beetroot ^7^	ud	0.48 (0.94) [<0.14–2.50]	5.45 (7.96) [0.10–20.50]	6.84 (12.84) [<0.09–31.25]	0.63 (0.96) [<0.35–2.25]	21.25 (33.98) [1.80–80.65]	2.31 (0.54) [1.35–3.00]	0.80 (0.84) [0.45–2.88]	2.46 (5.08) [<0.29–14.20]	16.76 (20.18) [1.20–47.80]
Broccoli ^7^	ud	0.93 (1.85) [<0.14–3.70]	119.42 (127.40) [6.80–302.50]	36.86 (42.95) [<0.09–98.95]	2.40 (0.56) [1.88–3.06]	173.32 (121.1) [72.00–326.38]	15.52 (8.31) [9.38–27.13]	5.27 (4.77) [1.13–10.25]	0.69 (0.80) [<0.29–1.50]	93.04 (62.71) [47.25–181.88]
Brussel sprout ^7^	ud	0.45 (0.78) [<0.14–1.35]	115.05 (174.71) [1.60–316.25]	37.39 (43.78) [1.31–86.10]	9.41 (4.20) [4.60–12.38]	252.58 (150.91) [114.31–413.56]	17.08 (7.97) [10.50–25.94]	2.97 (2.65) [<0.08–5.10]	10.59 (12.63) [<0.29–24.56]	166.58 (43.09) [119.50–204.06]
Carrot ^7^	ud	0.90 (1.60) [<0.14–3.69]	12.13 (16.88) [<0.06–41.25]	7.03 (9.26) [<0.09–17.50]	1.88 (1.93) [<0.35–4.38]	64.66 (83.96) [4.25–186.63]	5.52 (1.60) [3.10–7.50]	1.67 (0.86) [0.44–2.38]	5.39 (8.90) [<0.29–20.50]	23.35 (31.95) [<0.07–61.19]
Cauliflower ^7^	ud	0.52 (0.25) [0.38–0.80]	91.98 (110.97) [0.06–215.25]	32.22 (50.23) [0.94–90.15]	1.38 (2.40) [<0.35–4.15]	80.24 (69.17) [26.75–158.35]	21.27 (5.15) [17.44–27.13]	5.58 (0.84) [4.60–6.06]	21.58 (37.38) [<0.29–64.75]	46.23 (77.28) [0.31–135.45]
Celery ^7^	ud	0.33 (0.58) [<0.14–1.00]	58.29 (20.18) [35.50–73.88]	25.33 (21.94) [<0.09–38.38]	2.13 (0.87) [1.63–3.13]	93.17 (19.77) [70.50–106.88]	6.73 (1.29) [5.38–7.94]	1.46 (0.69) [1.00–2.25]	1.17 (1.02) [<0.29–1.88]	51.69 (13.23) [36.44–60.13]
Champignon ^7^	ud	6.73 (1.03) [5.60–8.15]	1.40 (3.07) [<0.06–6.90]	<0.09	0.52 (0.48) [<0.35–0.90]	1.93 (1.59) [0.45–4.45]	74.58 (18.71) [58.65–106.65]	2.10 (0.55) [1.40–2.65]	<0.29	38.56 (37.11) [0.50–85.20]
Fermented lupine brine ^10^	1	0.05	0.40	0.67	nd	13.14	2.90	5.48		0.21
Garlic ^7^	ud	1.75 (2.06) [<0.14–4.50]	8.46 (7.84) [<0.06–17.69]	3.04 (4.83) [<0.09–11.25]	0.69 (1.22) [<0.35–2.81]	67.65 (105.29) [4.25–249.44]	18.62 (9.21) [8.31–33.06]	6.44 (2.06) [3.94–9.40]	1.47 (2.51) [<0.29–5.80]	8.44 (7.75) [1.06–21.45]
Pepper ^7^	ud	2.48 (0.50) [2.00–3.10]	0.08 (0.15) [<0.06–0.30]	<0.09	0.88 (0.09) [0.75–0.95]	9.29 (3.17) [6.45–13.80]	1.21 (0.14) [1.10–1.40]	0.99 (0.11) [0.90–1.15]	<0.29	18.98 (2.92) [15.65–22.75]
Pickled caperberries ^11^	9		3.2 (3.1)	14.7 (17.2)		13.1 (8.5)		4.9 (4.4)		1.6 (2.6)
Pickled capers ^11^	8		nd	8.2 (6.7)		2.3 (1.3)		2.3 (2.4)		0.2 (0.6)
Pumpkin ^7^	ud	1.08 (1.29) [<0.14–2.75]	20.97 (0.76) [20.00–21.69]	29.58 (31.13) [2.88–73.94]	1.13 (1.44) [<0.35–3.00]	136.98 (54.51) [55.44–169.13]	8.68 (1.23) [7.06–9.75]	49.63 (34.89) [2.00–83.20]	4.88 (3.95) [<0.29–9.25]	62.61 (39.09) [20.06–111.69]
Radish ^7^	ud	0.32 (0.55) [<0.14–0.95]	14.18 (20.27) [0.88–37.50]	22.37 (22.04) [<0.09–44.06]	1.77 (0.93) [0.75–2.56]	32.08 (22.78) [6.38–49.80]	6.40 (2.92) [4.40–9.75]	0.93 (0.62) [0.45–1.63]	10.25 (11.38) [<0.29–22.50]	22.50 (11.33) [15.25–35.56]
Red cabbage ^7^	ud	3.76 (4.60) [0.85–9.06]	90.22 (61.65) [34.75–156.60]	32.00 (52.03) [0.44–92.05]	1.11 (1.56) [<0.35–2.90]	124.17 (140.70) [4.00–278.95]	10.75 (4.77) [6.25–15.75]	3.23 (0.64) [2.70–3.94]	9.90 (17.15) [<0.29–29.70]	59.65 (56.04) [0.19–111.50]
Sunchoke ^7^	ud	<0.14	4.50 (3.48) [1.50–8.31]	0.46 (0.79) [<0.09–1.38]	0.67 (1.15) [<0.35–2.00]	28.90 (18.15) [16.69–49.75]	7.83 (2.07) [5.88–10.00]	3.50 (0.22) [3.38–3.75]	<0.29	0.58 (0.71) [<0.07–1.38]
Tomato ^7^	ud	0.06 (0.11) [<0.14–0.19]	1.58 (1.00) [0.75–2.69]	1.65 (2.21) [0.15–4.19]	2.09 (2.47) [<0.35–4.81]	42.05 (29.97) [10.95–70.75]	3.79 (1.18) [2.50–4.81]	1.21 (1.02) [0.50–2.38]	1.21 (2.09) [<0.29–3.63]	8.34 (13.30) [0.45–23.69]
White cabbage ^7^	ud	3.14 (3.05) [<0.14–8.05]	35.76 (45.14) [<0.06–125.44]	55.60 (21.14) [32.55–83.81]	1.92 (0.95) [0.90–3.69]	190.59 (163.47) [57.50–524.63]	9.08 (2.48) [5.81–11.85]	2.55 (1.78) [0.69–5.38]	11.27 (5.89) [3.10–17.19]	60.69 (29.30) [29.05–105.13]
White turnip ^7^	ud	<0.14	4.57 (4.31) [1.13–10.70]	0.02 (0.03) [<0.09–0.06]	1.31 (2.63) [<0.35–5.25]	15.49 (12.96) [2.25–32.63]	6.38 (2.41) [4.31–9.69]	1.44 (1.37) [<0.08–3.00]	<0.29	16.26 (15.57) [<0.07–35.31]

The average amounts of the biogenic amines are given. Standard deviation is given in parenthesis, and the range is given in square brackets. Amounts are given in mg/kg unless otherwise stated. CAD: cadaverine; DOP: dopamine; HIS: histamine; NOR: noradrenaline; PHE: 2-phenylethylamine; PUT: putrescine; SER: serotonin; SPD: spermidine; SPM: spermine; TRP: tryptamine; TYR: tyramine; N.: number of samples examined; ud: undefined; nd: not detected; tr: traces. ^1^ Hornero-Mendez and Garrido-Fernandez [206] (in μg/mL); ^2^ Garcia-Garcia et al. [207]; ^3^ Moret et al. [194] (in mg/100 g fresh weight); ^4^ Jin et al. [203]; ^5^ Lee et al. [204]; ^6^ Cho et al. [202]; ^7^ Kang et al. [205]; ^8^ Tsai et al. [201] (in mg/100 g); ^9^ Shin et al. [208]; ^10^ Swider et al. [200]; ^11^ Kalac et al. [209]; ^12^ Tofalo et al. [210].

## Data Availability

All data related to this manuscript are presented in the form of tables and figures in the manuscript.
